# Evaluating environmental tobacco smoke exposure in a Group of turkish primary school students and developing intervention methods for prevention

**DOI:** 10.1186/1471-2458-7-202

**Published:** 2007-08-10

**Authors:** Hasan C Ekerbicer, Mustafa Celik, Ekrem Guler, Mehmet Davutoglu, Metin Kilinc

**Affiliations:** 1 Department of Public Health, Faculty of Medicine, Kahramanmaras Sutcu Imam University, Kahramanmaras, Turkey; 2 Department of Family Medicine, Faculty of Medicine, Kahramanmaras Sutcu Imam University, Kahramanmaras, Turkey; 3 Department of Pediatrics, Faculty of Medicine, Kahramanmaras Sutcu Imam University, Kahramanmaras, Turkey; 4 Department of Biochemistry, Faculty of Medicine, Kahramanmaras Sutcu Imam University, Kahramanmaras, Turkey

## Abstract

**Background:**

In countries like Turkey where smoking is highly prevalent, children's exposure to tobacco smoke is an important public health problem. The goals of this study were to determine the self-reported environmental tobacco smoke exposure status of primary school students in grades 3 to 5, to verify self-reported exposure levels with data provided from a biomarker of exposure, and to develop methods for preventing school children from passive smoking.

**Methods:**

The study was conducted on 347 primary school students by using a standard questionnaire and urinary cotinine tests. Children with verified ETS exposure were randomly assigned to 2 intervention groups. Two phone interviews were conducted with the parents of the first group regarding their children's passive smoking status and its possible consequences. On the other hand, a brief note concerning urinary cotinine test result was sent to parents of the second group. Nine months after the initial urinary cotinine tests, measurements were repeated in both groups.

**Results:**

According to questionnaire data, 59.9% of the study group (208 of 347) were exposed to ETS. Urinary cotinine measurements of children were highly consistent with the self-reported exposure levels (P < 0.001). Two different intervention methods were applied to parents of the exposed children. Control tests suggested a remarkable reduction in the proportion of those children demonstrating a recent exposure to ETS in both groups. Proportions of children with urinary cotinine concentrations 10 ng/ml or lower were 79.5% in Group I and 74.2% in Group II (P > 0.05).

**Conclusion:**

Self-reported ETS exposure was found to be pretty accurate in the 9–11 age group when checked with urinary cotinine tests. Only informing parents that their childrens' ETS exposure were confirmed by a laboratory test seems to be very promising in preventing children from ETS.

## Background

Exposure to ETS, also known as passive smoking, is a well-known health hazard in children. ETS is a real and substantial threat to child health, causing suffering and death throughout the world. The World Health Organization (WHO) has reported that almost half of the children in the world (nearly 700 million) are exposed to cigarette smoke and the majority of this exposure takes place at home [1,2].

The public health consequences of ETS exposure are enormous. ETS exposure increases children's risk of respiratory tract infections, otitis media, asthma, allergic disorder and the sudden infant death syndrome [3,4].

Most smokers are of child bearing age, leading to as many as 50% of children exposed in their homes [1,5-7]. Thus, reduction of ETS in infants and children is an important public health goal.

The WHO recommended legislation and education to protect children from ETS exposure [1]. However, few studies have demonstrated efficacious interventions. Obviously, getting smokers to quit smoking should protect children from ETS exposure [8]. However, Wahlgren and colleagues found that 67% of parents were unable to quit or reduce their asthmatic child's ETS exposure after physician advice [9]. Thus, something more than advice to quit is needed to achieve adequate protection.

The most important measures are restrictive legislation and control of price [10-12]. In addition, communication methods that target maternal behavior change might be relevant. However various studies can be found in the medical literature that show communication and counselling methods might be effective on maternal smoking behavior [13-16]. In these studies, the communication methods were focused on protecting the child from tobacco smoke, regardless of whether or not the mothers smoked. Persuading the mothers to give up smoking was not a primary goal. The outcome variables in these studies were urinary cotinine level [13], self-reported smoking [13-15], pulmonary function tests [14], and air measurements (carbon monoxide) [16]. In the medical literature to date, there are only a few studies on confirmation of self-reported exposure status of 9–11 year-old school children.

In this study, our purposes were (a) to determine environmental tobacco smoke exposure levels of primary school students by measuring urinary cotinine levels, (b) to verify children's self-reported exposure levels by comparing questionnaire data with data derived from urinary cotinine tests, and (c) to develop methods for preventing school children from ETS exposure.

## Methods

The study was conducted between September 2004 and September 2005 in 3 private primary schools in the city of Kahramanmaras, Turkey. Three hundred and forty-seven students in the third to fifth grades (9–11 years of age) of these schools were included into the study. After getting permission from Kahramanmaras City Education Department, all parents were informed about the study and their written consents were obtained.

The study was divided into two phases. In the first phase, students' exposure levels to ETS were determined by a standard questionnaire and urinary cotinine tests. The students were informed before administration of the questionnaire in their classrooms by one of the researchers and the teacher. The questionnaire consisted of 10 questions regarding demographic data (sex, grade), exposure status (number of smoking household members, number of daily exposed cigarettes, duration of exposure, outside exposure) and parents' educational status. Consistency of questionnaire data and urinary cotinine test results were also evaluated in this phase of the study. Twenty two students with (-) urinary cotinine test results contrary to their self-reports were excluded from the study. Similarly, 11 students with (+) test results who had reported no ETS exposure were not included into the second phase of the study. However, parents of these 33 students were informed about possible health risks of ETS by a phone interview. In the second phase of the study, 186 students with confirmed ETS exposure were randomly assigned to two groups. Parents of the first group (Group I) were interviewed by a psychologist trained in smoking addiction. Interviews were conducted by phone on two different occasions. During the interviews with the parents, four issues were emphasized: (a) ETS exposures were self-reported by their children and confirmed by a urine test; (b) consequences of exposure to ETS in children and how to protect them; (c) possible health risks for the smoker himself/herself; (d) where they can get help in quitting smoking. Parents of the second group (Group II) were informed by a brief note with the sentence: "Your child's exposure to tobacco smoke was detected by a urine test". The notes were signed by the parents and sent back. Urinary cotinine tests were repeated nine months after the first urine tests and approximately 7 months after the counselling sessions with the parents of Group I were completed.

### Urine analyses

The urine samples were collected during school time (except Mondays) before 12:00 pm without preservatives and kept at +4°C until use. An Immulite 2000 autoanalyser (DPC: Diagnostic Product Corporation, Los Angeles, USA. Cat no: L2KNM2) was used for nicotine metabolite measurement. Nicotine metabolite measurements were done by solid phase competetive chemiluminescent immunoassay and measured in ng/ml. Before testing all urine samples, 2 calibrators were used (4 times each). For quality control purposes, 2 control pools for 2 levels of cotinine (low and high) were used. The low control showed a total CV of 13.3% (mean 12.8, SD 1.7 ng/ml) and the high control showed a total CV of 6.2% (mean 47.1, SD 2.9 ng/ml). Same quality control procedures were used in the second stage of the study.

### Statistical analysis

Data were expressed as number of subjects and percentages. Statistical analyses were performed by using the Pearson correlation coefficient and Chi-square test. P values < 0.05 were considered statistically significant. All data were entered into and processed by SPSS version 11.0 for Windows statistical package (*SPSS Inc., Chicago, IL*).

## Results

Of 347 students, 208 (59.9%) reported ETS exposure at home. In response to the question "Which household member (or members) is smoking regularly at home?" 89 (42.7%) of the students pointed out both parents, 80 (38.4%) reported only their father, 26 (12.7%) reported 3 or more household members, and 13 (6.2%) reported only their mother. In the homes of 55.4% of the passive smoking children, two or more household members were smoking regularly. Children with self-reported ETS exposure addressed no exposure outside the house. Of these 208 students, 131 (62.9%) were being exposed to 1–3 cigarettes per day at home and 77 (36.1%) were being exposed to 4 or more. In reply to the question regarding daily duration of exposure: 128 (61.5%) responded 1–2 hours and 80 (38.5%) 3 or more hours. There was no significant correlation between number of cigarettes exposed to, daily duration of exposure and urinary cotinine levels (p > 0.05 for both).

The proportion of self-reported exposure did not differ significantly between sexes, grades or parents' educational status (p > 0.05) (Table 1).

**Table 1:** Comparison of some demographic characteristics of students according to their self-reported exposure status

	**Self reported exposure**		**P value**
		
	**Yes**	**No**	
**Sex (n = 347)**			
Male	124 (62.0%)	76 (38.0%)	>0.05
Female	84 (57.2%)	63 (42.8%)	
**Grade (n = 347**)			
3rd	66 (61.7%)	41 (38.3%)	
4th	79 (63.2%)	46 (36.8%)	>0.05
5th	63 (54.8%)	52 (45.2%)	
**Mother's education (n = 336)**			
Primary-Middle School	63 (57.2%)	47 (42.8%)	
High School	66 (62.3%)	40 (37.7%)	>0.05
University and over	78 (65.0%)	42 (35.0%)	
**Father's education (n = 341)**			
Primary-Middle School	12 (20.0%)	8 (40.0%)	
High School	79 (64.8%)	43 (35.2%)	>0.05
University and over	117 (58.5%)	83 (41.5%)	

**Figure 1:**
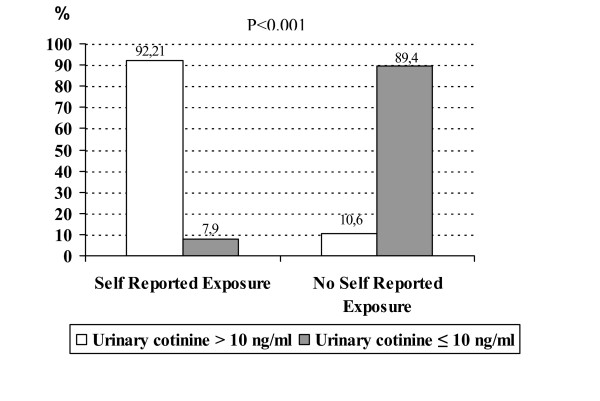
Distribution of students according to self-reported exposure status and urinary cotinine levels (n = 347).

None of the students was found to be an active smoker according to urinary cotinine test results. The highest urinary cotinine level mesured in both stages of the study was 81 ng/ml. Of the 208 students with self-reported ETS exposure, 10.6% of them (22) had urinary cotinine levels ≤10 ng/ml while 89.4% (186) had between 11–500 ng/ml. These proportions were 92.1% (128) and 7.9% (11), respectively, in 139 non-exposed students. The distribution of students according to self-reported exposure status and urinary cotinine levels are shown in Figure 1.

**Figure 2:**
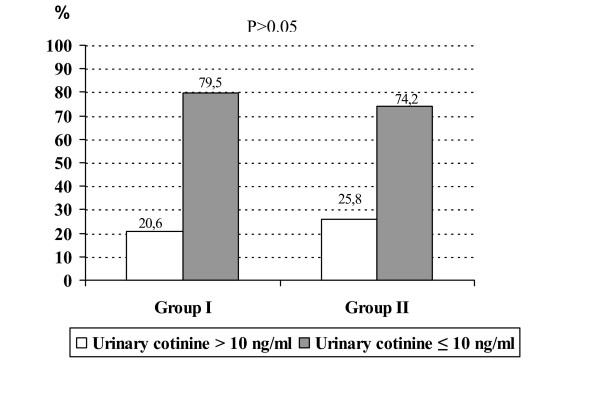
Comparison of two intervention groups nine months after the initial urinary tests.

Urinary cotinine tests were repeated to evaluate the effectiveness of both interventions at 9 months follow-up. In Group I, 74 students out of 93 (79.5%) had urinary cotinine levels below 10 ng/ml compared to 69 out of 93 (74.2%) in Group II. The percentages of children with urinary cotinine values equal to 10 ng/ml or less were statistically similar (p > 0.05) in both intervention groups (Figure 2).

## Discussion

In our study, 59.9% of the children were reported ETS exposure at home. In the United States the prevalence of children living in homes with a smoker has been estimated to be 43%, with state specific estimates of exposure at home ranging from 12% to 34% [5]. Thirty-five percent of children in the United States live in homes in which residents or visitors smoke on a regular basis [17]. Similarly, about 43% of Australian children [18], 33% of Canadian children [19], 41% of British children [20], 59.2% of the students in Mexico [21] and 89.0% of Turkish children [22] are exposed to environmental tobacco smoke.

In the present study, 89 (42.7%) of the students pointed out both parents smoking at home, while 80 (38.4%) reported only their father, 26 (12.7%) reported 3 or more household members and 13 (6.2%) reported only their mother. In a study conducted by Celik et al. [23], it was found that 12.6% of the mothers, 49.2% of fathers, and overall in 42.2% of the families, one or more persons smoked at home.

We found that mothers with higher education levels were more likely to smoke at home, although not statistically significant. In another study conducted in Kahramanmaras, Celik et al. [23] showed that rates of homes with at least one smoking household member and rates of smoking mothers were positively correlated with the educational levels of mothers. Moreover, in a study representing whole the Turkish population, it was reported that smoking more than 10 cigarettes was most common among women with at least a high school education (45%) [24]. The fact that studies in Western countries reveal a negative correlation between women's education levels and smoking status, unlike studies from Turkey, may be because of the conservative socio-cultural environment of the country [25,26].

Socioeconomic factors also are known to be related to cotinine levels. Parental education and family income both may be indicators of the prevalence of smoking in the community in which the child lives and plays [27,28]. The effect of maternal smoking on child salivary cotinine level has been reported to be greater than the effect of paternal smoking in England and Wales, especially with high levels of cigarette consumption [29]. Housing characteristics have also been previously described as being associated with cotinine levels, with smaller homes predicting higher levels among smoke-exposed children [28]. In our study, the proportion of self-reported exposure did not differ significantly between sexes, grades or parents' educational status, as well as between urinary cotinine levels and number of cigarettes exposed to and the daily duration of exposure. In contrast to our study, it has been reported by 3 different studies that the number of cigarettes that parents smoke is a major determinant of salivary cotinine concentration in children [27,28,30]. This contradiction may be attributable to the choice of method in measuring cotinine. Consistent with our results, Smyth et al. [31] have reported that salivary cotinine was more closely related to family smoking behavior than urinary cotinine concentrations.

Most estimates of the exposure of infants and young children to tobacco smoke are based on adult reports. Self-reports have the advantage of low cost and ease of administration, but raise questions of reliability and validity. Brunekreef et al. [32] emphasize that underreporting of ETS exposure by parents of study children varies, and may depend on the instrument used, population studied, age, and symptom status, underlining the need for questionnaire validation in specific study settings. In addition to urinary cotinine tests, we used a questionnaire to assess subjects' ETS exposure levels. Besides those with 33 (9.5%) of the students, all of the others cotinine test results were consistent with the self-reports. In interpreting our results, the age group of our subjects and the fact that they are private school students must be considered.

In our study, mean urinary cotinine levels of two intervention groups measured nine months after the initial urinary tests were similar, and in both groups, urinary cotinine levels of the majority of the students were below 10 ng/ml (Figure 2). We suggest that a brief intervention incorporating feedback of children's urinary cotinine levels via a letter would display a similar positive effect on parents' attitude on restricting smoking at home as feedback plus intensive counselling. In another study with a similar design to ours, Wakefield et al. [33] reported no significant change in smoking habits of parents between intervention (written and verbal feedback about child's urinary cotinine level) and control (usual advice about smoking) groups. Their study differed from ours in that children in their intervention group were predominantly from low-income families, and 69.5% of mothers were smoking at home. Three other studies concerning children's exposure to ETS at home have revealed that counselling and advice, without feedback on children's urinary cotinine levels, did not change the children's exposure to ETS [27,34-36]. In this context, Hovell et al.'s study [15] is an exception, in which authors reported that an intervention involving more intensive counselling was associated with significant decreases in children's exposure to ETS. The results of this study were obtained with a group of low-income families, in which families were paid as an incentive to participate.

## Limitations of the study

One of the limitations of our study is a lack of a control group made up of passive smokers, whose parents were informed about their children's self reported exposure to ETS but not informed about urinary cotinine test results. However, forming three subgroups within the passive smokers would result small sample sizes. Also, the high consistency of self reported exposure and urinary cotinine concentrations, and the assumption that the parents belonging to a high socioeconomic group have at least a basic level of knowledge about the harms of ETS, encouraged us to complete the study without any further control group.

Conducting the study only in private schools in order to reach a socioeconomically homogeneous group of students makes it difficult to generalize our results to all 9–11 year-old students in the city or the country. However, we believe that providing data on the consciousness of a highly educated group of parents regarding ETS in a country where more than 60 percent of men smoke is of value.

Although there are more specific tests for quantitating human exposure to ETS, urinary cotinine tests were preferred due to limited funding.

## Conclusion

The prevalence of passive smoking was fairly high in the study group. Self-reported ETS exposure status of 9–11 year-old students were consistent with their urinary cotinine levels. Informing parents that their children's ETS exposure was confirmed by a laboratory test had a positive effect in preventing them from home ETS exposure. Further studies with a similar design should be carried out in socio-economically different population groups. We also think that investigation of families who develop effective policies in reducing the ETS exposure of their children will be benefical. If further studies confirm our findings, carrying out urinary cotinine tests routinely in the presence of self-reported ETS exposure may be considered for this age group.

## Competing interests

The author(s) declare that they have no competing interests.

## Authors' contributions

HCE designed the paper, performed the analyses, and is the primary author. MC designed the paper, provided data and commented on early drafts. EG commented on early drafts. MD provided data and commented on early drafts. MK provided data and commented on early drafts. All authors read and approved the final manuscript.

## Pre-publication history

The pre-publication history for this paper can be accessed here:


